# Factors responsible for the persistence of Hypothyroidism among Pakistani Women

**DOI:** 10.12669/pjms.40.1.8446

**Published:** 2024

**Authors:** Mariyah Hidayat

**Affiliations:** 1 Mariyah Hidayat University of Lahore, Lahore, Pakistan

**Keywords:** Climate Change, Hypothyroidism, Thyroid Disrupting Chemicals, Environmental Pollution, Pesticides

## Abstract

Substantial change in climate over the years in South Asia is directly affecting the diet and health of the population. It is important to analyze the aftermath of this change and its impact on the thyroid status. In recent years, a complex interplay of the changing climate with the environmental, geographical and dietary factors has contributed to the continued prevalence of hypothyroidism in Pakistani women. To study the influence of various factors which might be aggravating hypothyroidism in the female population of Pakistan, a thorough search of literature was conducted of various databases including Google Scholar and PubMed. Google as a search engine was also explored. This included both interventional and observational studies, published in English, from the year 1950 onwards uptil June 2023. It also included WHO website and local news clips about the awareness campaigns on iodine deficiency over the past years. All studies conducted on females to diagnose hypothyroidism, with both positive and negative outcomes were included in this study.

The factors contributing to hypothyroidism among the female population in Pakistan encompass the effects of climate change, both direct and indirect, topographical factor, indiscriminate use of hazardous pesticides, presence of chemical contaminants in food and water, and a lack of awareness among the public and healthcare professionals about the condition’s symptoms and management.

## INTRODUCTION

Around the globe, two hundred million people including South Asians suffer from thyroid disorders,^1^,^2^ mostly due to under functioning of the thyroid gland. In spite of alarming number of cases, there is limited information about the reasons for variability between human thyroid functions across countries, races, gender, and age. Generally, women are five to eight times more likely than men to develop thyroid diseases throughout the world, and one in every eight women will develop a thyroid disorder during her lifetime.^3^ It is not scientifically known why women are so vulnerable to thyroid dysfunctions in all parts of the world, but multiple factors are responsible for the persistence of hypothyroidism in women living in Pakistan; a developing country located in South Asia with rising cases of thyroid disorders.^4^

To reduce the risk of hypothyroidism, a holistic framework is required to better understand the causes of under functioning thyroid gland in Pakistan - a country most vulnerable to this condition on account of its geographical location, unstable socioeconomic conditions and changing climate.

### Literature Search Strategy

To study the influence of various factors which might be aggravating hypothyroidism in female population of Pakistan, a thorough search of literature was conducted of various databases including Google Scholar and PubMed. Google as a search engine was also explored. This included both interventional and observational studies, published in English, from the year 1950 onwards uptil June 2023. It also included WHO website and local news clips about the awareness campaigns on iodine deficiency over the past years. All studies conducted on hypothyroid females with both positive and negative outcomes were included in literature search.

## FACTORS CONTRIBUTING TO HYPOTHYROIDISM – LITERATURE REVIEW

### 1. Direct influence of climate change

Environmental and climate change in South Asia is directly affecting the diet and health of the population. It is important to analyze the aftermath of this change and its impact on the thyroid status. Pakistan is highly vulnerable to climate change which is not only an environmental issue but also a rising health issue for the country.^5^ As with the changing climate in South Asia, the climate of Pakistan has been adversely affected over the past few decades,^6^ which has had a significant impact on the nutritional quality of food. This has directly affected the health of the people and is expected to further deteriorate it with passing years. Changes in environmental temperature may alter the serum concentration of thyroid hormones and its metabolism.^7^

### 2. Indirect influence of Climate Change

Although there is limited direct scientific evidence to suggest that climate change itself is a significant factor in the aggravation of hypothyroidism, however, it can indirectly influence certain factors that might impact thyroid health. These factors include iodine availability in soil and water, and its impact on air quality and drinking water.

### Lack of iodine in soil and water

About 40% of the world population are at risk of iodine deficiency,^8^ in which the thyroid gland may enlarge resulting in goiter. Hypothyroidism is the most prevalent form of thyroid disease in Pakistan, the reason documented so far being lack of iodine in the diet.^9^ Poverty is perhaps responsible for lack of iodine, as in terms of the number of poor, about 35 million out of the total of 47 million people fall below the poverty line in Pakistan.^10^ In addition to malnutrition, lack of awareness about nutritional value of micronutrients in the diet and unhealthy eating habits may also be leading to a hypo functioning thyroid gland.

### Impact of climate change on drinking water resources

Change in climate has a profound impact on drinking water resources, with subsequent consequences on thyroid health and quality of life. Pakistan has recently witnessed a deadly aftermath of environmental change in the form of excessive rainfall and floods during monsoon in the months of July and August, 2022.^11^ Additionally, intense heat waves during summers have caused the glaciers on the mountain peaks to melt over the years, dwindling fresh water reserves at the basin, and leading to intense flooding of the Indus Valley River System,^12^ which is further affecting the quality of diet. Persistent floods in the coming years may further affect the chemical quality of water, resulting in further disruption of hormones and adversely affecting the functioning of the thyroid gland.

### Impact on quality of air

Air pollutants especially particulate matter in outdoor air have become one of the biggest threats to the health of Pakistani population.^13^ These are well-known human carcinogens which are increasingly associated with adverse effects on the thyroid gland, including disturbance of thyroid function and lowering its capacity to synthesize hormones.^14^-^16^ Pakistan is ranked as the world’s fourth most polluted country and all of its 225 million people live in areas where the annual average air pollution level exceeds the WHO guideline, whereas 99% percent live in areas where pollution level exceeds Pakistan’s own air quality standards.^17^ Moreover, increased atmospheric carbon dioxide levels due to global warming may be another source of impact on the levels of micronutrients in the crops and vegetation.^18^,^19^

Although a significant relationship has been reported between air pollution and thyroid function in limited samples in some regions of the world,^20^ no study so far has addressed this association in the general population of Pakistan, where the citizens are regularly exposed to unhealthy air. The daily exposure to air pollutants can significantly impact health of the thyroid gland, especially in females,^21^ as they have been reported to be more vulnerable to air pollution and hormonal disruptions.^22^ Estrogen and progesterone might be contributing to this sex disparity, which needs further investigation.

### Geographical factor

The topography of this country might be another reason for prevalence of hypothyroidism, as it comprises barren mountains and valleys with poor road connectivity which is unfavorable for transportation of goods to remote areas, depriving the outreach areas of commodities. Different studies on thyroid disorders have been conducted in the northern areas of Pakistan including the province of KPK over the years and goiter has been the most frequently observed condition, mostly in children and females. One commonly stated reason to justify the number of cases diagnosed with goiter is perhaps the geographical location of this province comprising of steep valleys which are not favorable for farming. In I972, Chapman et al. was the first person to describe the prevalence of thyroid disorders in the general population in mountainous northern areas of Pakistan.^23^ Later, in the year 1981, the prevalence of palpable goiter in Kalam, a mountainous region located in the province of KPK was reported as 21.18% in the 5-11 years of age group.^24^ In 1998, in the same province, 59.2% iodine deficiency in one localized area was reported,^25^ whereas the school children examined clinically and diagnosed with goiter in a study conducted in 2002 in northern areas of Pakistan was 39.27%.^26^ Later, in 2014, a survey was conducted in the public schools of Peshawar (KPK), which showed 59% prevalence of initial stage goiter in children of under 10 years of age.^27^ Another study conducted in 2016 on the population of Peshawar (KPK) concluded that thyroid diseases are more common in females as compared to males.^28^ All of this statistical data highlight the alarming number of cases of hypothyroidism in the province of KPK over the years. However, cases of hypothyroidism in females have been reported in the provinces of Punjab and Sind too, who are neither iodine deficient nor pregnant. A survey conducted in 2017 revealed the prevalence of subclinical hypothyroidism in the population of Karachi, with a frequency of 13.6% in females compared to only 9.2% in males.^29^ To investigate a link between subclinical hypothyroidism and serum iodine levels, a study was conducted on individuals who resided in iodine sufficient areas and those who dwelled in low iodine areas in some parts of the world, and the results showed that greater number of cases of hypothyroidism were prevalent in populations on iodine sufficient diet compared to those on a diet poor in iodine.^30^ Similarly, the province of Punjab has fertile lands ideal for farming and dairy which are the major dietary sources of iodine and therefore, it is more likely that in addition to iodine deficiency, there are other causes of hypothyroidism in that region. As for the province of Baluchistan and Gilgit Baltistan, there is no recorded data or evidence of any research work conducted in these areas about the state of thyroid health of the population in the past or the present.

### Indiscriminate use of hazardous pesticides

There is growing evidence that environmental contaminants can disrupt endocrine functions,^31^,^32^ which are impurities that are either introduced by humans or occurring naturally in water, air or soil. In developing agricultural countries, pesticides are the most frequently found environmental contaminants acting as thyroid disruptors, affecting the hypothalamic-pituitary-thyroid axis at several levels to decrease the serum concentration of thyroid hormones.^33^ In Pakistan, where agriculture is the prime occupation in rural communities, there is overwhelming evidence of indiscriminate use of hazardous pesticides by farmers without taking adequate precautions. Disregarding protective measures is posing an increased health risk, including harm to the health status of the thyroid gland.^34^,^35^

### Presence of Chemical contaminants in food and water

The contamination of soil with chemicals has led to poor quality of vegetation which is another source of toxicants for the thyroid gland. Other than pesticides, there are many other sources of contaminants polluting water and soil in Pakistan.^36^ Frequent flooding and unchecked waste disposal from the industries into the river water is another major source of environmental chemicals. Chemical contaminants reported to be found in water and soil that may alter the thyroid status of individuals include cadmium, arsenic, mercury, lead, copper, sulfate and sodium,^37^ but there has been no initiative taken to measure the amount of these chemicals in river water across the country, which is the main source of drinking water. Especially after the heavy floods witnessed this year, its suspected consequences on the quality of drinking water might be alarming, if properly investigated.

There is no system in place for inspecting and measuring the presence of chemical contaminants in food and water. These slowly developing health hazards due to recurrent episodes of flooding and rain might be causing a significant impact on the thyroid status of population across the country which may further increase in magnitude in the coming years.

### Inadequate access to health information by the public

Access to health information by the public plays a vital role in spreading awareness to help control the disease. Different studies on thyroid disorders have been conducted locally by researchers in the northern areas of Pakistan over the past years and goiter has been the most frequently observed condition, mostly in children and women.^23^-^28^ However, this observation has never been shared with the general public. There are innumerable women suffering from this condition who are not even aware of being hypothyroid, as they perhaps have never been tested. In remote areas, they do not even know whether such condition even exists that is harmful to their health, and so the number of cases of hypothyroidism keep on increasing. As the majority of the population live in rural areas, they are not aware of the risk factors or consequences of thyroid disorders. This may be the reason why many of them often revert to homeopathic and traditional remedies when diagnosed with thyroid disease, instead of consulting the doctor and taking proper medication to treat their symptoms. However, symptoms are barely noticeable in many cases. Moreover, health issues due to hypothyroidism tend to develop slowly, often over a number of years.

In the year 1994, Pakistan formally adopted the universal salt iodization program and launched a mass campaign to alleviate hypothyroidism by encouraging the public to consume iodized salt in their daily food intake.^25^ Remarkably, this landmark awareness campaign ran successfully for years, and the reason why it gained momentum was taking the public into confidence and apprising them about the hazards of low iodine intake. As a result of this campaign years ago, the majority of the people in Pakistan still consume iodized salt. However, despite this massive campaign, hypothyroidism persists among Pakistani women. In spite of awareness campaigns run by the government to consume iodized salt, the outcomes have never been measured.

### Lack of awareness among health professionals

The signs of hypothyroidism can be so subtle at times that they may go undetected by the clinicians. Often at times, due to high patient turnover and workload in government hospitals, doctors are so overwhelmed with work that they may overlook checking the thyroid status of the patients, even when all symptoms are present. These cases often go unchecked and undocumented, and if diagnosed by chance, they are not taken notice of by the health authorities.

Even though there is no data available to compare the past and present number of cases of hypothyroidism to measure the impact of iodized salt intake, various studies conducted in different regions of the country still show high prevalence of this disease, especially in females.^28^-^30^ Across the country, in spite of rich data available in many laboratories and hospitals, there is limited information about the total number of cases of hypothyroidism, as after iodine intervention program, the government never made an effort to gather and record data on the number of cases, to measure the impact of the program. The current source of information about the rising cases of hypothyroidism is mostly research articles in local health journals written by health professionals based on small scale self-funded studies, but this data is neither accessible to the public nor understandable to them.

Overall, the underlying causes of hypothyroidism or its regional distribution across the country has not been explored and there is no data available on the nation-wide cases and distribution of this disease. Government officials, policy makers and health professionals should prioritize revisiting this area of health the way they did many years ago by introducing iodized salt intake. They should reassess and re-plan timely the measures to protect thyroid health of the population.

### Limitations

There are certain limitations of this study which includes lack of quality data. The studies conducted across the country over the years are not only limited but also lack adequate sample size which does not ensure generalizability. Moreover, limited access to comprehensive databases could potentially impact the reliability of the conclusions drawn from this review. While the review aims to identify causes contributing to hypothyroidism, establishing causal relationships between certain factors and the condition can be complex. Many of the identified causes require more rigorous study designs such as longitudinal studies or randomized controlled trials. Moreover, this review has not explained the patterns in climate change over the years, which would need further exploration.

## CONCLUSION

The factors contributing to hypothyroidism among the female population in Pakistan encompass the effects of climate change, both direct and indirect, geographical factor, indiscriminate use of hazardous pesticides, presence of chemical contaminants in food and water, and a lack of awareness among the public and healthcare professionals about the condition’s symptoms and management.

### Recommendations

Health professionals need to review the thyroid health of the female Pakistani population amidst all the predisposing factors. Given below are few recommendations for the readers, policy makers and health care professionals. The following measures may make a substantial difference in controlling hypothyroidism and its outcomes;

1. Conducting public awareness campaigns to educate individuals about hypothyroidism, its symptoms, risk factors, and the importance of early diagnosis and treatment.
2. Training of healthcare professionals to enhance their knowledge and diagnostic skills related to thyroid disorders, enabling them to identify and manage hypothyroidism more effectively.
3. Supplementation of diverse and balanced diets through nutrition programs that include iodine-rich foods to improve overall thyroid health.
4. Regulation of the inadvertent use of pesticides by farmers and agricultural industry.
5. Monitoring the quality of food and drinking water.
6. Collaborating with environmental agencies to address the impact of climate change on iodine availability, air quality, and water resources, focusing on strategies to mitigate these effects.
7. Encouraging research initiatives to further investigate the intricate connections between environmental factors, such as climate change and pesticide use, and thyroid health outcomes.
8. Foster collaboration between healthcare professionals, environmental experts, policymakers, and community leaders to develop holistic strategies that address the multifaceted causes of hypothyroidism.
9. Integrate thyroid health education into school curricula, raising awareness among the younger generation and promoting healthy habits from an early age.

## References

[1] Keestra S, Hogqvist Tabor V, Alvergne A (2020). Reinterpreting patterns of variation in human thyroid function:An evolutionary ecology perspective. Evol Med Public Health.

[2] ОІРябуха. The untapped potential of the thyroid axis. Editorial (2013). Lancet Diabetes Endocrinol.

[3] Walker GL (2022). Why women are more susceptible to thyroid disease?.

[4] Mishra AK, Agarwal A, Parameswaran R, Tin S, Fernando R, Alhajri K Thyroid Surgery in South Asia. InEndocrine Surgery.

[5] Malik SM, Awan H, Khan N (2012). Mapping vulnerability to climate change and its repercussions on human health in Pakistan. Glob Health.

[6] Chaudhry Q (2017). Climate change profile of Pakistan. The Asian Development Bank.

[7] Sarne D (2016). Effects of the Environment, Chemicals and Drugs on Thyroid Function, MDText. com. Inc, South Dartmouth, MA, USA.

[8] Editorial. The untapped potential of the thyroid axis (2013). The Lancet Diabetes and Endocrinology.

[9] Hussain Z, Elahi S Undetected hypothyroidism and its anesthetic implications. Anaesthesia, Pain &Intensive Care.

[10] Khan H, Inamullah E, Shams K (2009). Population, environment and poverty in Pakistan:linkages and empirical evidence. Environment, Development and Sustainability.

[11] Otto FE, Zachariah M, Saeed F, Siddiqi A, Kamil S, Mushtaq H (2023). Climate change increased extreme monsoon rainfall, flooding highly vulnerable communities in Pakistan. Environmental Research:Climate.

[12] Chaudhary A, Clark A (2022). Melting Himalayan Glaciers Are Making Pakistan's Floods Worse. Bloomberg, Asia Edition.

[13] Air quality in Pakistan.

[14] Darbre PD (2018). Overview of air pollution and endocrine disorders. Int J Gen Med.

[15] Plunk EC, Richards SM (2020). Endocrine-disrupting air pollutants and their effects on the hypothalamus-pituitary-gonadal axis. Int J Mol Sci.

[16] Zhang Y, Wang K, Qin W, Jin C, Song Y, Jia P, Wang S, Song Y, Ning Y, Li L (2021). Six Air pollutants associated with increased risk of thyroid nodules:A study of 4.9 million Chinese adults. Fronti Endocrinol.

[17] Ramasamy DK (2020). Enchanted improvements in air quality across India-A study from COVID-19 lockdown perspective. Adalya J.

[18] Ebi KL, Ziska LH (2018). Increases in atmospheric carbon dioxide:Anticipated negative effects on food quality. PLoS Med.

[19] Anjum MS, Ali SM, Subhani MA, Anwar MN, Nizami AS, Ashraf U, et al (2021). An emerged challenge of air pollution and ever-increasing particulate matter in Pakistan;a critical review. J Hazardous Mater.

[20] Izic B, Husejnovic MS, Caluk S, Fejzic H, Kundalic BS, Custovic A (2022). Urban Air Pollution Associated with the Incidence of Autoimmune Thyroid Diseases. Med Arch.

[21] Janssen BG, Saenen ND, Roels HA, Madhloum N, Gyselaers W, Lefebvre W, Penders J, Vanpoucke C, Vrijens K, Nawrot TS (2017). Fetal thyroid function, birth weight, and in utero exposure to fine particle air pollution:a birth cohort study. Environmental health perspectives.

[22] Arias-Ortiz NE, Icaza-Noguera G, Ruiz-Rudolph P (2018). Thyroid cancer incidence in women and proximity to industrial air pollution sources:A spatial analysis in a middle size city in Colombia. Atmospheric Pollution Res.

[23] Chapman J, Grant I, Taylor G, Mahmud K, Ul-Mulk S, Shahid, M (1972). Endemic goitre in the Gilgit agency, west Pakistan. Philos Trans R Soc London B Biol Sci.

[24] Paindakhel S (1981). Goitre in North of Kalam. J Pak Med Assoc.

[25] Khattak RM, Khattak MK, Ittermann T, Volzke H (2017). Factors affecting sustainable iodine deficiency elimination in Pakistan:A global perspective. J Epidemiol.

[26] Khan A, Khan MMA, Akhtar S (2002). Thyroid disorders, etiology and prevalence. J Med Sci.

[27] Sarwar S (2014). Urinary iodine levels in residents of Azad Jammu and Kashmir. J Rawal Med Coll.

[28] Attaullah S, Haq BS, Muska M (2016). Thyroid dysfunction in Khyber Pakhtunkhwa, Pakistan. Pak J Med Sci.

[29] Khan MA, Ahsan T, Rehman UL, Jabeen R, Farouq S (2017). Subclinical Hypothyroidism:Frequency, clinical presentations and treatment indications. Pak J Med Sci.

[30] Farebrother J, Zimmermann MB, Andersson M (2019). Excess iodine intake:sources, assessment, and effects on thyroid function. Ann New York Acad Sci.

[31] Diamanti-Kandarakis E, Bourguignon JP, Giudice LC, Hauser R, Prins GS, Soto AM (2009). Endocrine-disrupting chemicals:an Endocrine Society scientific statement. Endocr Rev.

[32] Leemans M, Couderq S, Demeneix B, Fini JB (2019). Pesticides With Potential Thyroid Hormone-Disrupting Effects:A Review of Recent Data. Front Endocrinol (Lausanne).

[33] BabićLeko M, Gunjača I, Pleić N, Zemunik T (2021). Environmental factors affecting thyroid-stimulating hormone and thyroid hormone levels. Int J Mol Sci.

[34] McLachlan JA, Simpson E, Martin M (2006). Endocrine disrupters and female reproductive health. Best Pract Res Clin Endocrinol Metab.

[35] Anaduaka EG, Uchendu NO, Asomadu RO, Ezugwu AL, Okeke ES, Ezeorba TP (2023). Widespread use of toxic agrochemicals and pesticides for agricultural products storage in Africa and developing countries:Possible panacea for ecotoxicology and health implications. Heliyon.

[36] Buha A, Matovic V, Antonijevic B, Bulat Z, Curcic M, Renieri EA, Tsatsakis AM, Schweitzer A, Wallace D (2018). Overview of Cadmium Thyroid Disrupting Effects and Mechanisms. Int J Mol Sci.

[37] Sohail R, Yasmin H, Tasneem N, Khanum Z, Sachdeve PS, Pal SA (2021). The Prevalence of Subclinical Hypothyroidism During Early Pregnancy in Pakistan:A Cross-Sectional Study. Cureus.

